# Pilot experience of multidisciplinary team discussion dedicated to inherited pulmonary fibrosis

**DOI:** 10.1186/s13023-019-1256-5

**Published:** 2019-12-03

**Authors:** Raphael Borie, Caroline Kannengiesser, Laurent Gouya, Clairelyne Dupin, Serge Amselem, Ibrahima Ba, Vincent Bunel, Philippe Bonniaud, Diane Bouvry, Aurélie Cazes, Annick Clement, Marie Pierre Debray, Philippe Dieude, Ralph Epaud, Pascale Fanen, Elodie Lainey, Marie Legendre, Aurélie Plessier, Flore Sicre de Fontbrune, Lidwine Wemeau-Stervinou, Vincent Cottin, Nadia Nathan, Bruno Crestani

**Affiliations:** 1 Service de Pneumologie A, DHU FIRE, Centre de Référence (Site Constitutif) Maladies Pulmonaires Rares, APHP, Hôpital Bichat, 46 rue Henri Huchard, 75877 Paris, CEDEX 18 France; 2 INSERM, Unité 1152, Université Paris Diderot, Paris, France; 3 Laboratoire de Génétique, APHP, Hôpital Bichat, Paris, France; 4 Université Paris Diderot, Paris, France; 5 Département de Génétique, U.F. de Génétique moléculaire, APHP, Sorbonne Université, Inserm U933, Hôpital Trousseau, Paris, France; 6 APHP, Hôpital Bichat, Service de Pneumologie B, DHU FIRE, Paris, France; 7 Service de Pneumologie et Soins Intensifs Respiratoires, Centre de Référence (Site Constitutif) Maladies Pulmonaires Rares, CHU Dijon-Bourgogne, Dijon, France; 8 Service de Pneumologie, Hôpital Avicenne, Centre de Référence (Site Constitutif) Maladies Pulmonaires Rares, APHP, Bobigny, France; 9 APHP, Hôpital Bichat, Service d’Anatomopathologie, Paris, France; 10 Service de Pneumologie Pediatrique, Hôpital Trousseau, Filière RespiFil, APHP, Paris, France; 11 APHP, Hôpital Bichat, Service de Radiologie Paris, Paris, France; 12 APHP, Hôpital Bichat, Service de Rhumatologie, Paris, France; 13 Centre des Maladies Respiratoires Rare, Respirare® Centre Hospitalier Intercommunal de Créteil, Inserm, Unité 955, Equipe 5, Université Paris-Est, Faculté de Médecine, Creteil, France; 14 Laboratoire de Génétique, APHP, Hôpital Henri Mondor, Paris, France; 15 Laboratoire d’hématologie, APHP, Hôpital Robert Debré, Paris, France; 16 APHP, Service d’hépatologie, Hôpital Beaujon, Clichy, France; 17 APHP, Service d’hématologie, Hôpital St Louis, Paris, France; 18 Service de Pneumologie, Centre de Référence (Site Constitutif) Maladies Pulmonaires Rares, CHU de Lille, Lille, France; 19 Coordonnateur, OrphaLung, Centre national de référence des maladies pulmonaires rares, Service de Pneumologie, Hôpital Louis Pradel, UMR754, Université Claude Bernard Lyon 1, Lyon, France

**Keywords:** Interstitial pulmonary fibrosis, Telomerase, Surfactant, TERT, Familial; multidisciplinary discussion

## Abstract

**Background:**

Genetic testing is proposed for suspected cases of monogenic pulmonary fibrosis, but clinicians and patients need specific information and recommendation about the related diagnosis and management issues. Because multidisciplinary discussion (MDD) has been shown to improve accuracy of interstitial lung disease (ILD) diagnosis, we evaluated the feasibility of a genetic MDD (geneMDD) dedicated to the indication for and interpretation of genetic testing. The geneMDD group met monthly and included pediatric and adult lung specialists with ILD expertise, molecular and clinical geneticists, and one radiologist. Hematologists, rheumatologists, dermatologists, hepatologists, and pathologists were also invited to attend.

**Results:**

Since 2016, physicians from 34 different centers in 7 countries have participated in the geneMDD. The medical files of 95 patients (53 males) have been discussed. The median age of patients was 43 years [range 0–77], 10 were ≤ 15 years old, and 6 were deceased at the time of the discussion. Among 85 analyses available, the geneMDD considered the rare gene variants pathogenic for 61: 37 variants in telomere-related genes, 23 variants in surfactant-related genes and 1 variant in *MARS*. Genetic counseling was offered for relatives of these patients. The geneMDD therapeutic proposals were as follows: antifibrotic drugs (*n* = 25), steroids or immunomodulatory therapy (*n* = 18), organ transplantation (*n* = 21), watch and wait (*n* = 21), or best supportive care (*n* = 4).

**Conclusion:**

Our experience shows that a dedicated geneMDD is feasible regardless of a patient’s age and provides a unique opportunity to adapt patient management and therapy in this very rare condition.

## Introduction

The central role of multidisciplinary discussion (MDD) in the diagnostic algorithm of interstitial lung disease (ILD) was recently highlighted by the 2018 ATS/ERS/JRS/ALAT recommendations for the diagnosis of idiopathic pulmonary fibrosis (IPF) [1]. ILD-specific MDD should include expert respiratory physicians and at least one radiologist and one histopathologist with specific expertise in ILD; experienced rheumatologists and immunologists are of utmost help in difficult cases [2]. MDD is the worldwide standard of care in ILD centers [2–5].

The field of monogenic pulmonary fibrosis has made great progress in the last 10 years, raising specific issues that should be addressed by a specialized team [6]. Approximately 30% of patients with a familial history of pulmonary fibrosis are carriers of mutations in telomere-related genes (TRGs), surfactant-related genes or other rare genes [6]. Monogenic ILD could also arise in an apparently sporadic context because of incomplete penetrance and variable expressivity or recessive inheritance. For instance, lung fibrosis associated with a mutation in a TRG is frequently associated with specific hematological or hepatic diseases that may be at the forefront [7] and raise specific diagnostic and therapeutic issues [8–11]. Genetic disorders of surfactant dysfunction have been recognized as underlying causes of respiratory disease in neonates and children as well as adults and requires a close interaction with pediatricians with dedicated expertise [12]. Finally, the genetic diagnosis in this field is particularly difficult and requires a specific expertise that is not available in many ILD centers [3, 6, 13].

To offer the expertise required for the diagnosis, interpretation of genetic data and treatment of patients suspected to have a genetic form of lung fibrosis, we have set up a web-based multicenter genetic MDD (geneMDD) dedicated to all suspected or confirmed cases of inherited lung fibrosis. Here we describe the geneMDD set-up and our retrospective analysis of the impact of the geneMDD in terms of pulmonary and genetic diagnosis, disease management and genetic counseling for cases discussed to date in the geneMDD.

## Methods

### The geneMDD

The geneMDD was created in September 2016 and has met monthly ever since. It is chaired by a respiratory physician (RB) and includes at least one geneticist (molecular or clinical), one pediatrician with specific expertise in ILD, and one chest radiologist. When needed, a pathologist, rheumatologist, dermatologist, hepatologist, immunologist, hematologist and psychologist could also attend.

Patients with ILD of suspected or known genetic origin are proposed for discussion by their ILD physician. A standardized form, including a pedigree, is filed before the meeting and presented by the referring physician. Chest high-resolution CT images and histology reports are reviewed during the MDD. The referring physician can come to Bichat hospital or connect by visioconference sharing his screen to show the requested images as well as the pedigree.

### Inclusion criteria

Patients in this study represent consecutive patients referred to the geneMDD from September 2016 to October 2018. Any patient with suspected inherited pulmonary fibrosis, without age limitation, could be discussed. A genetic testing was not required for the discussion, but most patients had at least *TERT* or *TERC* sequencing results available [7]. Our actual proposal for a genetic analysis is the presence of familial pulmonary fibrosis, a specific syndrome suggestive of an heritable pulmonary fibrosis such as telomere syndrome, or cryptogenic pulmonary fibrosis before age 50 [6]. The geneMDD was offered to all patients with a variant of class 3 or more evidenced during that period. Patients could also be discussed on the request of the referring physician in case of negative results in a patient with highly suggestive heritable pulmonary fibrosis (e.g., young age and extra-pulmonary disease and > 2 ILD cases in the family) [7].

Patients could be deceased at the time of the geneMDD, and those cases were presented to discuss the genetic counseling. In that situation, the age at death was considered for the age at presentation.

### geneMDD meeting

During the geneMDD, clinical data, chest CT scan and lung histological pattern were reviewed and classified according to the 2018 ATS/ERS/JRS/ALAT statement for IPF and the 2013 ATS/ERS classification of idiopathic interstitial pneumonias [1, 14]. Chest CT scans were initially classified according to the 2011 ATS/ERS/JRS/ALAT Statement and were reclassified according to the latter classification in light of the geneMDD description [15]. The geneMDD provided a written conclusion, including a diagnosis; a suggestion for further diagnostic procedures, such as a surgical lung biopsy; and a treatment strategy, including evaluation for lung, liver or bone-marrow transplantation, antifibrotic therapy, steroids and immunomodulators, watch and wait, or best supportive care.

Genetic and functional analysis findings, when available, were reviewed, and genetic variants were classified according to the American College of Medical Genetics and Genomics guidelines and the European Society for Human Genetics recommendations [16]. For the variants of unknown significance (VUS), we considered variants with 1 moderate criteria and 3 supporting criteria for pathogenicity as a working diagnosis of damaging VUS (VUSD) [7, 16]. For each case, a genetic conclusion was proposed by the geneticist: pathogenic variant (class 4 or 5), VUSD, VUS (class 3), benign variant (class 2) or no variant identified. Benign variants usually do not appear in the genetic report. Complementary analysis coud be offered: functional analysis (e.g., telomere length, surfactant secretion in transfected cell lines or interferon signature, as described [7, 17, 18]), familial investigation, segregation study or extension of the genetic analysis (e.g., next-generation sequencing [NGS] panel or whole-exome sequencing [WES]). According to the genetic conclusion, genetic counseling could be proposed to the affected patient and relatives [6]. A survey was performed in January 2019 to evaluate the follow-up of the geneMDD proposals.

All patients signed informed consent for genetic analysis, including for research purposes. The clinical charts of the patients were collected on a standardized and anonymous form. This study was approved by the local ethics committee (CPP Ile de France 1, no. 0811760). All data are available on request.

## Results

### Patient characteristics

**Fig. 1:**
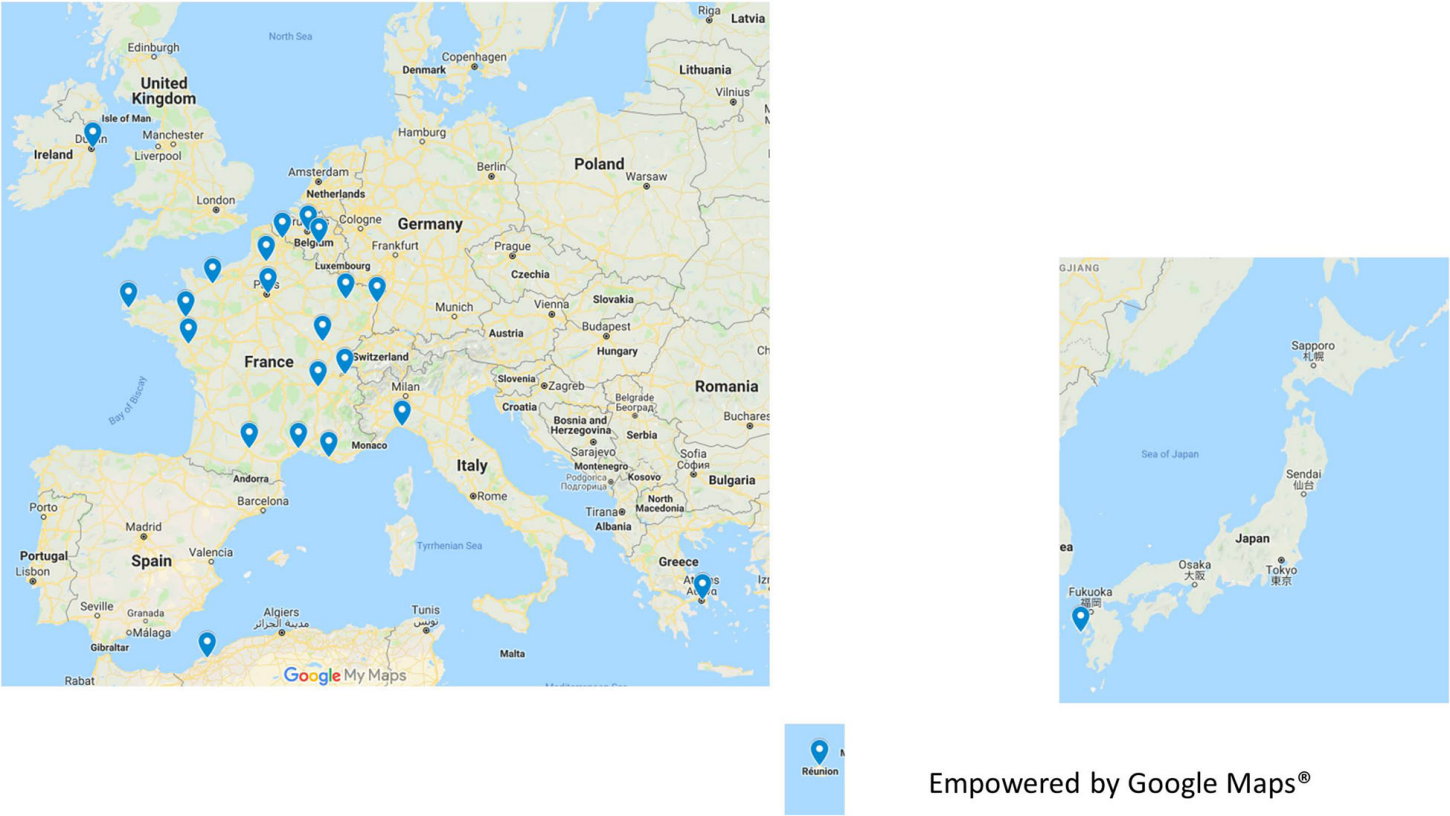
Overall, 34 different interstitial lung disease (ILD) centers from 7 different countries took part in the genetic multidisciplinary discussion (geneMDD) up to October 2018 (empowered by Google map®)

From September 2016 to October 2018, the geneMDD was held 18 times, and 34 different ILD centers from 7 different countries participated (France, Algeria, Belgium, Greece, Italy, Ireland and Japan; Table 1, Fig. 1). Overall, 95 patients (53 males) from 83 families were discussed, with a mean of 5.2 patients [range 2–12] discussed per session. The median age of the patients was 43 years [range 0–77]; 6 patients were deceased at the time of the geneMDD.

**Table 1:** Characteristics of the centers and main characteristics of the patients discussed at the genetic multidisciplinary discussion (geneMDD)

Number of participating centers	34
Number of countries	7
Number of patients discussed	95
Number of families	83
Patient age (years) (median [range])	43 [0–77]
Male	53 (56%)
CT pattern (*N* = 85)	
UIP or probable UIP	22 (26%)
Indeterminate for UIP	32 (37%)
Alternative to UIP	23 (27%)
No ILD	8 (9%)
Histology (*N* = 21)	
UIP or probable UIP	8 (38%)
Alternative diagnosis	9 (43%)
Unclassifiable	4 (19%)
Pulmonary function tests (median [range])	
FVC (% of predicted value)	68 [24–135]
DLCO (% of predicted value)	52 [13–108]

Data are n (% of available data) unless indicated. (%). *FVC* forced vital capacity, *DLCO* diffusing capacity of lung for CO, *UIP* usual interstitial pneumonia, *ILD* interstitial lung disease

### Genetic analysis and counseling

Indications for genetic testing were familial pulmonary fibrosis (*n* = 53, 55%), specific syndrome (*n* = 30, 32%, including 27 [28%] with telomere syndrome and 3 [3%] with brain, lung thyroid syndrome), cryptogenic ILD before age 50 (*n* = 43, 45%), or asymptomatic relative (*n* = 13, 13%). Some patients had several indications for genetic analysis.

Genetic analyses were not available for 10 patients at the time of the geneMDD (5 ongoing, 5 not yet done). Among the 85 cases with available genetic analyses, 58 had a targeted genetic analysis including *TERT* or *TERC* sequencing or only one gene such as *NKX2–1* analyzed; 24 had NGS panel testing including TRGs, and 3 had WES results available. *TERC* and *TERT* were initially the only genes tested in familial pulmonary fibrosis or telomere syndrome; other TRGs such as *RTEL1* or *PARN* were later included in the NGS panel.

**Fig. 2:**
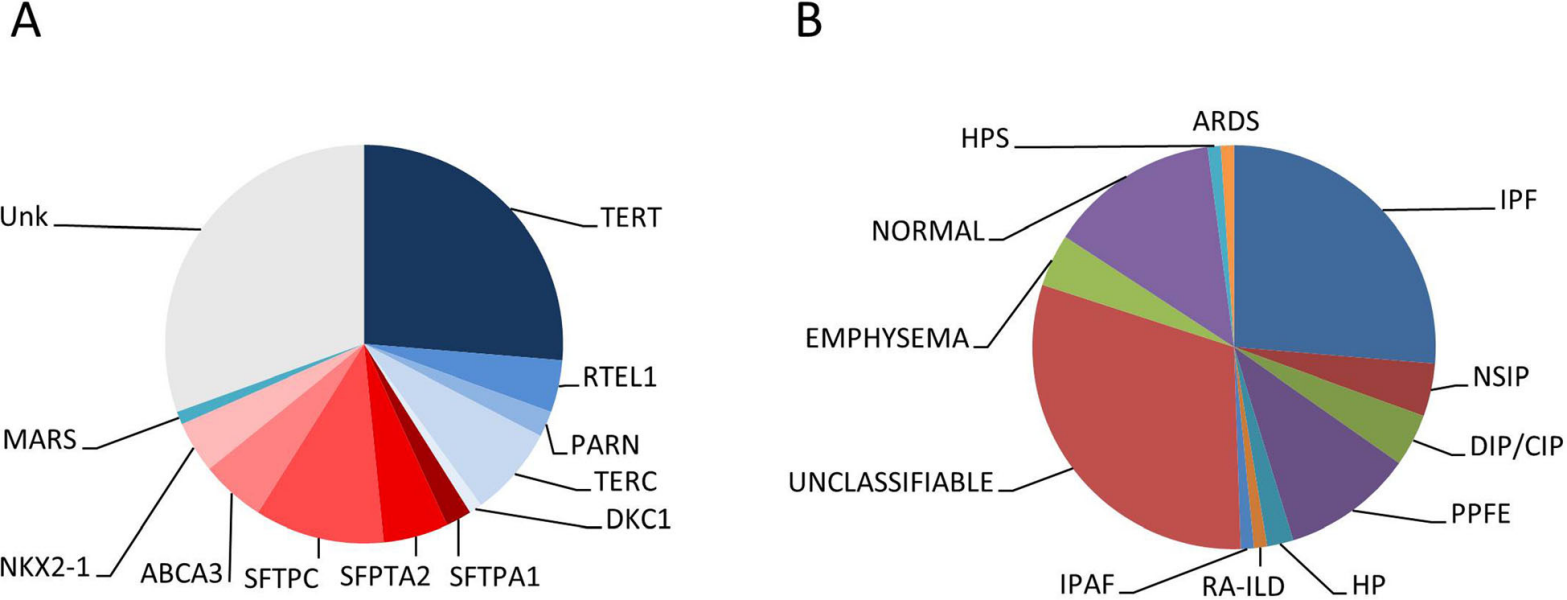
(**a**) Genetic variants (variants of unknown significance [VUS] or pathogenic) discussed during the geneMDD, (**b**) Pulmonary diagnoses proposed by the geneMDD. IPF, idiopathic pulmonary fibrosis; NSIP, non-specific intersititial pneumonia; DIP, desquamative intersitial pneumonia; CIP, cellular interstitial pneumonia; PPFE, pleuro-parenchymal fibroelastosis; HP, hypersensitivity pneumonitis; RA-ILD, rheumatoid arthritis interstitial lung disease; IPAF, interstitial pneumonia with auto-immune features; HPS, hepatopulmonary syndrome; ARDS, Acute respiratory distress syndrome; Unk, unknown

Before the geneMDD genetic analysis, rare monoallelic or biallelic variants were identified in 66 of the 85 (77%) patients analyzed, in accordance with the dominant or recessive inheritance. The variants included 22 VUS (class 3) and 44 pathogenic or likely pathogenic variants (class 4 and 5) (Fig. 2) [3, 5]. A rare variant within one TRG was identified in 39 cases (59%): *TERT* (*n* = 25, 37%), *TERC* (*n* = 7, 11%), *RTEL1* (*n* = 4, 6%), *PARN* (*n* = 2, 3%), and *DKC1* (*n* = 1, 1%). A rare variant within a gene of the surfactant pathway was identified in 26 cases (30%): *SFTPC* (*n* = 10, 15%), *SFTPA1* or *SFTPA2* (*n* = 7, 11%), *ABCA3* (*n* = 5, 8%), *NKX2–1* (*n* = 4, 6%) (Fig. 2). One patient carried a previously reported *MARS* mutation [19]. No case of digenic inheritance was considered in this series although we envision that in the era of next generation sequencing, whole exome sequencing and pan genome analyses, the number of patients with more than one rare variant will be growing.

After geneMDD file review, all variants initially considered pathogenic or likely pathogenic were retained as pathogenic (*n* = 44), 17 of 22 VUS were considered a VUSD [7], and 5 were still considered a VUS (Table 2). Additional evaluation was proposed for 39 patients (45%): WES or targeted NGS (*n* = 18, 21%); familial screening (*n* = 14, 15%); functional analysis (*n* = 16, 17%), including telomere length measurement (*n* = 7, 7%); surfactant analysis (*n* = 9, 9%); or interferon signature analysis (*n* = 3, 3%). The suggested analyses were done for 28 patients so far (72%).

**Table 2:** Pre- and post-geneMDD diagnosis

	Pre-geneMDD	Post-geneMDD
Pulmonary diagnosis		
IPF	**27 (28%)**	**25 (26%)**
Unclassifiable	**34 (36%)**	**29 (30%)**
Alternative ILD diagnosis	**18 (19%)**	**22 (23%)**
No ILD	**16 (16%)**	**18 (19%)**
Genetic diagnosis		
VUS	**22**	**5**
VUSD or pathogenic	**44**	**61**

*VUS* variant of unknown significance, *VUSD* working diagnosis of damaging variant

Bold data signifies Data ate N (%)

**Fig. 3:**
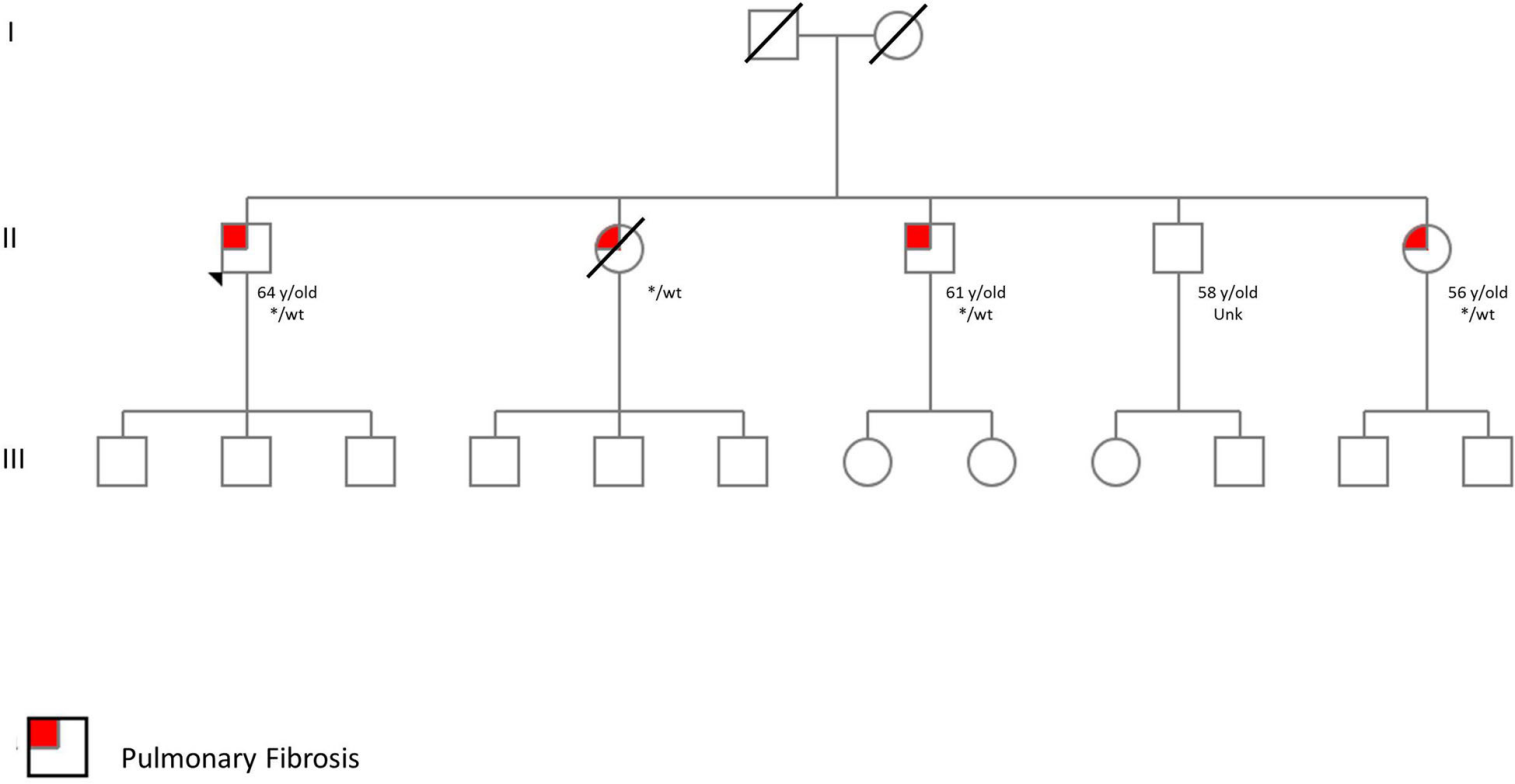
Pedigree of a family including 4 siblings with pulmonary fibrosis and heterorozygous carriers of a *TERT* mutation (c.2516C > T, p.Thr839Met, wild type (wt)/*). Individual II,4 refused the clinical and genetic evaluation. The geneMDD proposed genetic analysis for all children of generation III, which is currently ongoing

Additionally, the referring physician informed 61 patients (52 families) with overt disease that a presymptomatic genetic diagnosis for their relatives was recommended by the geneMDD. For 48 patients of childbearing age, a favorable opinion in principle was issued in the event of a request for prenatal diagnosis in a context of particular clinical gravity associated with pathogenic mutations. In January 2019, screening had been performed for 37 families (71%) (Fig. 3).

### Pulmonary diagnosis

**Fig. 4:**
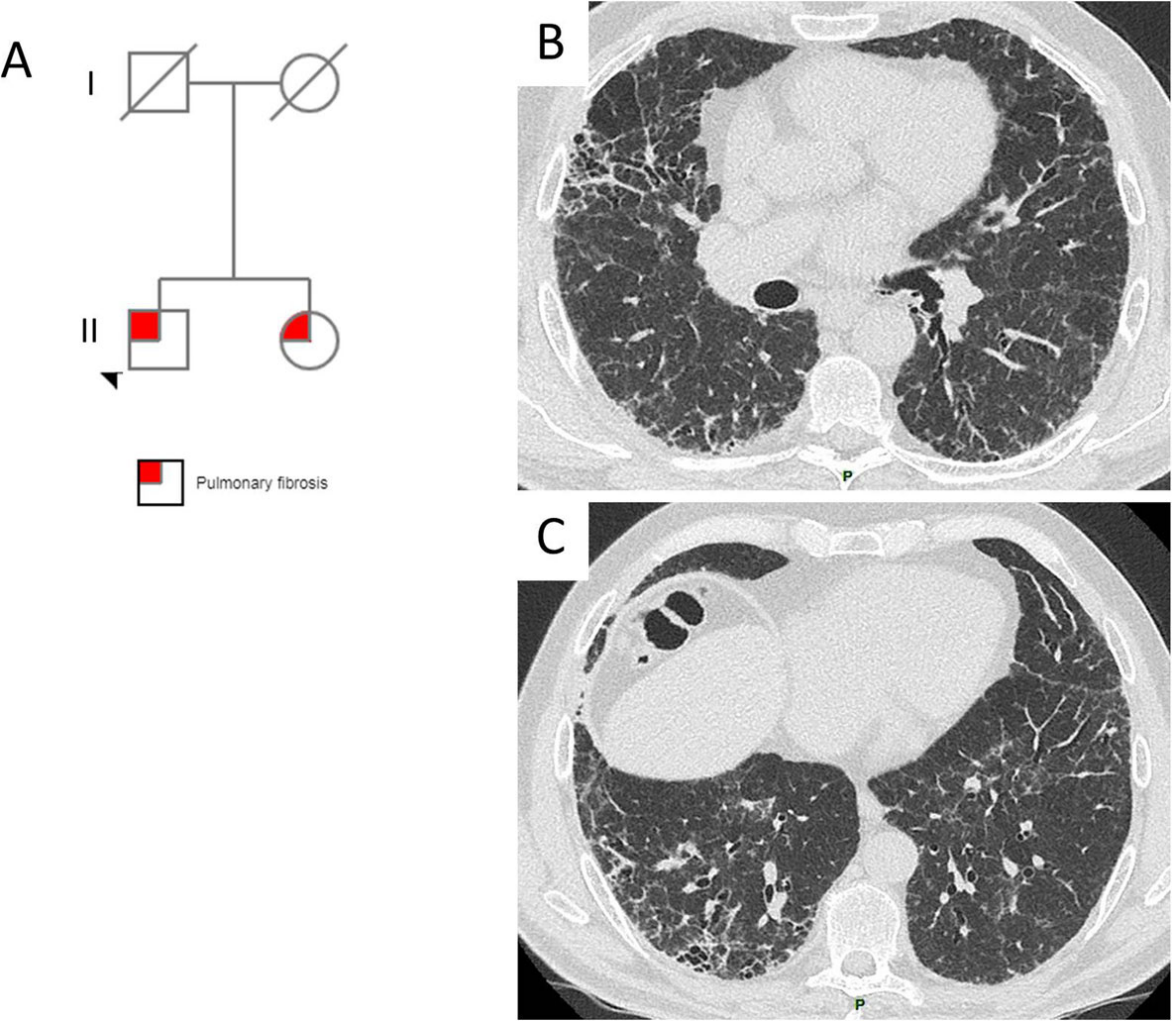
(**a**) A 64-year-old non-smoking man with familial pulmonary fibrosis and no extra-pulmonary manifestation. (**b**, **c**) The CT scan pattern was considered usual interstitial pneumonia (UIP). Genetic analysis revealed a heterogeneous *TERT* mutation (c.3216G > A, p.Trp1072*), classified as pathogenic, in both siblings. Genetic counselling was proposed for the relatives. Antifibrosing therapy was offered along with lung transplantation screening for the proband

**Fig. 5:**
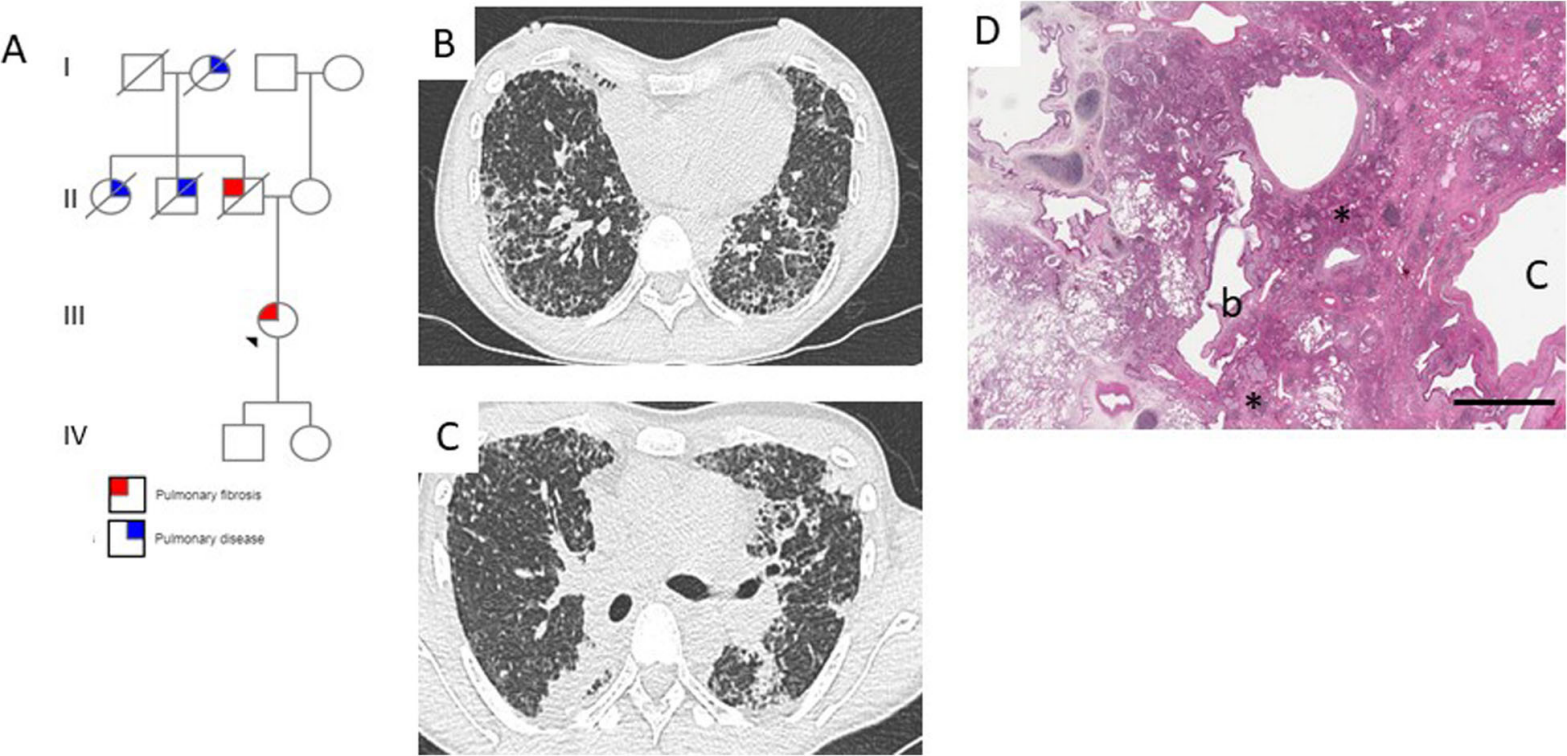
(**a**) A 44-year-old non-smoking woman with rheumatoid arthritis and familial pulmonary fibrosis. (**b**, **c**) The CT pattern was considered indeterminate for UIP and not suggestive of a specific diagnosis. Genetic analysis revealed a heterozygous *SFTPA2* mutation (c.532G > A, p.Val178Met) classified as pathogenic, and genetic counseling was proposed for the relatives. A double lung transplantation was proposed and performed in April 2017. **d** Histology of lung transplant tissue was considered indeterminate for UIP: patchy fibrosis with both subpleural and centrilobular fibrosis with dense inflammatory infiltrates (*).b: bronchiole, C: subpleural cyst. Hematoxylin Eosin Saffron stain, bar = 3000 μm

**Fig. 6:**
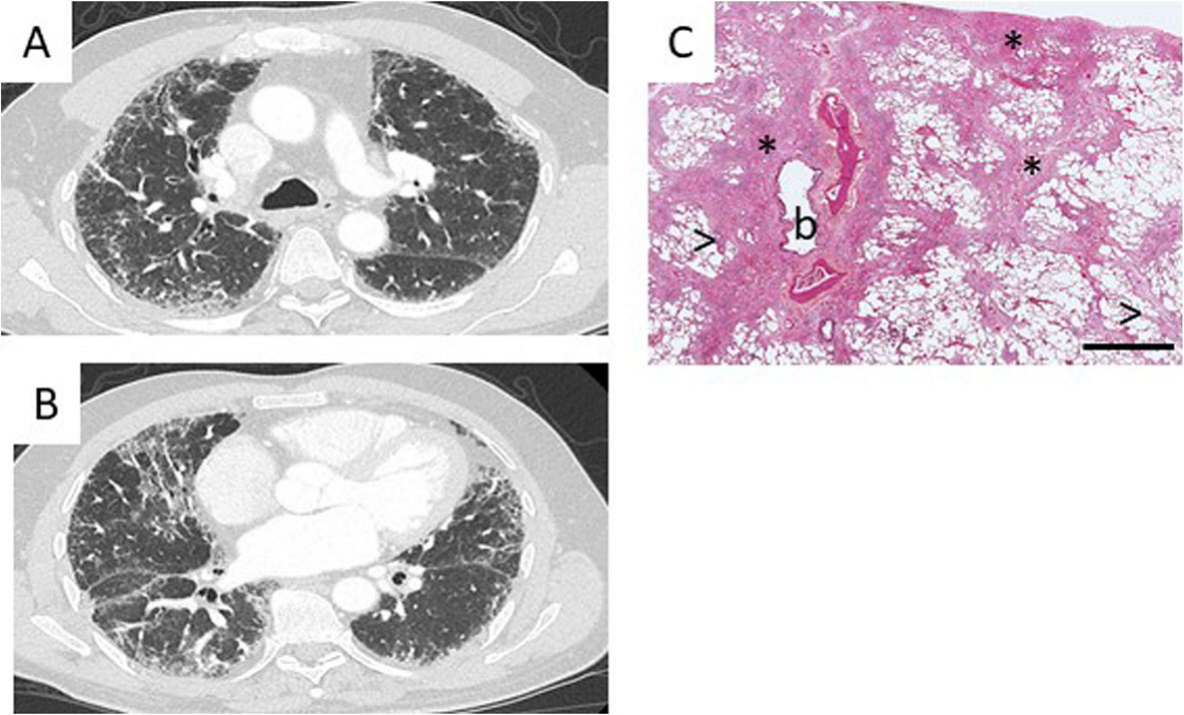
A 57-year-old patient with macrocytosis and liver steatosis. **a**, **b** The CT pattern was considered indeterminate for UIP, not suggestive of a specific diagnosis. Genetic analysis revealed a heterozygous *TERC* mutation (r.235C > G) classified as pathogenic, and genetic counseling was proposed for the relatives. Lung transplantation was proposed and performed in August 2018. **c** Histology of the lung transplant tissue was considered indeterminate for UIP: patchy fibrosis with both subpleural and centrilobular fibrosis (*) with dense inflammatory infiltrates and fibroblastic foci (>). (*), b: bronchiole. Hematoxylin Eosin Saffron stain, bar = 3000 μm

A CT scan was available for review in 85 cases (89%). The CT scan did not show any ILD in 8 (9%) patients. In the other cases, the CT pattern observed was definite or probable usual interstitial pneumonia (UIP) in 22 (26%); indeterminate for UIP in 32 (17%, including 5 previously classified as possible UIP and 27 without suggested specific diagnosis); or suggestive of an alternative diagnosis to UIP in 23 (27%). For these 23 patients, the CT pattern suggested a diagnosis of pleuro-parenchymal fibro-elastosis (PPFE, *n* = 11, 13%), desquamative interstitial pneumonia (DIP, *n* = 3, 4%), non-specific interstitial pneumonia (NSIP, *n* = 7, 8%) and hypersensitivity pneumonitis (HP, *n* = 2, 2%). The pattern was not suggestive of a specific diagnosis for 27 (32%) patients, mainly because of extensive ground-glass opacities and/or cysts (Figs. 4, 5 and 6).

Histology was available for 21 patients. UIP was the most frequent pattern (*n* = 9, 42%), followed by NSIP (*n* = 2, 10%), PPFE (*n* = 2, 10%), HP (*n* = 1, 5%), DIP (*n* = 1, 5%), and cellular interstitial pneumonia (*n* = 1, 5%). In five cases, the histological pattern remained unclassifiable (Figs. 5 and 6).

Before the geneMDD, the diagnosis was IPF for 27 patients (28%), and the geneMDD confirmed the diagnosis for 25/27 (93%) (Table 2 and Fig. 2). The pulmonary diagnosis was modified by the geneMDD for only 10 (10%) patients: for 7 patients, a diagnosis of unclassifiable pulmonary fibrosis before the geneMDD was reclassified as PPFE (*n* = 3), working diagnosis of IPF (*n* = 2) or no ILD (*n* = 2); conversely, for 3 patients, a diagnosis of IPF was reclassified as PPFE (*n* = 2) and unclassifiable pulmonary fibrosis (*n* = 1). After the geneMDD, the most frequent diagnoses were IPF (*n* = 25, 26%), unclassifiable pulmonary fibrosis (*n* = 29, 31%, including 24 patients without available histology − 10 patients having predominant ground glass opacities- and 5 with available histology from surgical lung biopsy), no ILD (*n* = 18, 19%; including 13 patients without normal CT scan, 1 with emphysema, 1 with hepato-pulmonary syndrome or 3 with bronchiolitis), and PPFE (*n* = 10, 10%). In addition, a diagnostic surgical lung biopsy was proposed for 4 patients and eventually performed for 3 of them. The histology was probable UIP (*n* = 1), unclassifiable fibrosis (*n* = 1) and DIP (*n* = 1).

In total, 41 patients required specific extra-pulmonary evaluation, for hematologic abnormalities (*n* = 20, 49%), liver abnormalities (*n* = 13, 32%), or rheumatologic disorders (*n* = 7, 17%) (Table 3). Hematological diagnoses were dysmyelopoiesis (*n* = 8), myelodysplasia (*n* = 4), toxic aplasia (*n* = 1), aplastic anemia (*n* = 1), refractory anemia with blast excess (*n* = 1), acute myeloid leukemia (*n* = 1), and isolated macrocytosis (*n* = 2). Two patients were considered free of hematological disease but had a familial history of acute leukemia. Including the results of 6 liver biopsies, the liver diseases were hepatic cytolysis of unknown etiology (*n* = 2), sinusoidal distension (*n* = 1), liver cirrhosis (*n* = 6), venoocclusive disease (*n* = 1), regenerative nodular hyperplasia (*n* = 1), and steatosis (*n* = 1). One patient was considered free of hepatological disease but reported a familial history of liver cirrhosis.

**Table 3:** Extra-pulmonary manifestations discussed by the geneMDD

Extra-pulmonary manifestations	
Total	41 (43%)
Hematological	20 (21%)
Hepatic	13 (14%)
Rheumatological	7 (7%)
Neurological^a^	6 (6%)
Immunological	5 (5%)
Dermatological^a^	2 (2%)
Ophthalmological^a^	2 (2%)
ENT^a^	2 (2%)

*ENT* ear nose and throat. ^a^ Specialists not attending the GeneMDD

### Treatment

A therapeutic strategy was offered to all living patients (*n* = 89): antifibrotic therapy (*n* = 25, 28%); watch and wait policy (*n* = 21, 23%); evaluation for lung transplantation (e.g. for the *MARS* mutation carrier, *n* = 20, 22%) and liver transplantation (*n* = 1); immunomodulatory therapy (*n* = 18, 20%), including steroids (*n* = 10), inhaled granulocyte-macrophage colony-stimulating (inhaled GM-CSF, *n* = 3), macrolides (*n* = 2), danazol (*n* = 2), hydroxychloroquine (*n* = 1), and statins (*n* = 1); and best supportive care (*n* = 4, 4%). According to the previous received treatment and the CT pattern the following treatment was offered to all living patients with unclassifiable fibrosis (*n* = 28): antifibrotic therapy (*n* = 7); watch and wait policy (*n* = 4); evaluation for lung transplantation (*n* = 3); immunomodulatory therapy (*n* = 9), including steroids (*n* = 7), inhaled GM-CSF (*n* = 3), hydroxychloroquine (*n* = 1), and statins (*n* = 1); and best supportive care (*n* = 3). Inhaled GM-CSF was offered to 3 patients with alveolar proteinosis superimposed with unclassifiable pulmonary fibrosis: 1 with *MARS* mutation and 2 brothers without any identified known mutation. Among all 64 patients for whom the geneMDD proposed medication, 63 (93%) eventually received it.

## Discussion

Here we report the results of the first genetic MDD dedicated to patients with ILD of suspected genetic origin. A total of 95 patients from 34 centers in 7 countries were discussed, which highlights the need for such a specific MDD and the unique experience we could acquire. Indeed, the geneMDD determined that 61 patients were carriers of a pathogenic mutation, which allowed for genetic counseling, performed for 71% of them. Moreover, the geneMDD suggested a specific therapy for 64 patients according to the pulmonary and extra-pulmonary diagnoses and the genetic conclusion; in 93% of the cases, the referring physician followed the geneMDD proposals.

With the increasing number of genetic variants identified in ILD patients, genetics expertise seems needed in the daily practice of ILD centers. From a technical viewpoint, genetic analysis methods are rapidly evolving and each technique has its own advantages and pitfalls. Moreover, the analysis of data generated by these techniques can be difficult. For instance, none of the TRG is the site of a recurrent mutation, and new genetic variants are continually being identified [20–22]. The genetic conclusion can therefore be difficult [10].

TRG mutations were the most frequent category evaluated during the geneMDD (59%). Patient carriers of TRG mutations also frequently present hematological and hepatic disease, so the presence of a hematologist and hepatologist with specific expertise is required for a thorough discussion of these cases [7, 9, 23, 24]. Because of evidence of anticipation in these families, a discussion with pediatricians was the rule when young adults with children were being discussed [17, 25]. The surfactant gene mutations were the second most frequent category of genes identified during the geneMDD supporting the presence of pediatricians.

Moreover, other specialists could participate in and be required by the geneMDD for specific cases. For instance, *NKX2–1* mutations are frequently associated with thyroid and neurological disorders, which require specific expertise [26]. Obviously, except for at least a requirement for one ILD specialist and one geneticist, other specialists were not required for the whole duration of the geneMDD. Indeed, the list of patients to discuss was prepared before the meeting to combine specific issues to discuss (pediatric, hematological or hepatic etc.)

Videoconferencing is relevant for an efficient meeting [3]. It allows for discussing at the same time different individuals from a single family living in different geographical areas, comparing the respiratory and extra-respiratory phenotypes, and adopting a coordinated and homogeneous approach for all family members. All ILD meeting will not include genetic evaluation, but videoconferencing allows every center to access genetic expertise for patients with suspected heritable pulmonary fibrosis. Conversely, with new clinical information, the geneticist was able to propose a diagnosis of VUSD after the geneMDD.

The geneMDD report includes the limitations for the diagnosis and therapeutic proposals and references any trial that could be proposed to the patient. Evidence available for these patients are currently limited. From a therapeutic point of view, no therapeutic trial dedicated to patients with genetic lung fibrosis supports any evidence-based therapeutic decisions. Post-hoc analysis of the ASCEND and CAPACITY trials indicated that pirfenidone slows the decline of lung function in patients with a TRG mutation [27]. Danazol has been tested in patients with a TRG mutation and hematological abnormalities, but the data concerning the lung in that study are very limited [8]. A retrospective study of pirfenidone efficacy in patients with a *TERT* or *TERC* mutation did not demonstrate a beneficial effect of pirfenidone on lung function decline in these patients [28]. Several retrospective series have reported the outcome of lung transplantation in ILD patients with *TRG* mutations and noted a specific hematological risk and possible reduced survival [9–11, 29, 30].

The geneMDD has several limitations because it actually relies on the referring physicians to volunteer to discuss their patient, inducing a selection bias. We now systematically offer for discussion the files of patients for whom a genetic variant is identified in our lab, though some centers did not discussed their cases in geneMDD and some cases were not proposed to the geneMDD during the first 2 years of operation. This approach is of particular importance when the variant is classified as a VUS. In such cases, only additional non-routine analyses such as telomere length, telomerase activity, or other functional studies could decipher their pathogenicity [16]. Moreover, the geneMDD insists on better characterizing all members of a family because the segregation study is an important point for a genetic conclusion [16]. Lastly it was not required to send the CT scan and the pathogical samples before the geneMDD. The radiologist (MPD) and the pathologist (AC) analyzed some of the CT scan and histological samples only during the geneMDD. Indeed, we have to assume that a double reading could reclassify some patients.

## Conclusion

We suggest that a valuable geneMDD should include at least an ILD specialist, a geneticist, a pediatrician, and one chest radiologist, and a web-based conference system with excellent imaging transmission. A specific report need to be given after the MDD. A dedicated secretary is important to collect the forms, data, and CT scan before the meeting, to send weblink, codes and solve technical issues during the meeting, and to complete, send and safely store the report for each patient after the meeting. However, our experience demonstrates that the geneMDD is feasible and offers the expertise for adequate management of genetic forms of pulmonary fibrosis. We believe that the geneMDD should be the standard of care for patients with suspected or confirmed genetic ILD, although it may be limited to an expertise center.

## Data Availability

All data are available on request.

## Abbreviations

CT: Computed tomography
DIP: Desquamative interstitial pneumonia
geneMDD: genetic multidisciplinary discussion
HP: Hypersensitivity pneumonitis
ILD: Interstitial lung disease
IPF: Idiopathic pulmonary fibrosis
MDD: Multidisciplinary discussion
NGS: Next-generation sequencing
NSIP: Non-specific interstitial pneumonia
PPFE: Pleuro-parenchymal fibro-elastosis
TRGs: Telomere-related genes
UIP: Usual interstitial pneumonia
VUS: Variants of unknown significance
VUSD: Working diagnosis of damaging variant
WES: Whole-exome sequencing
